# Adequacy of Maternal Iron Status Protects against Behavioral, Neuroanatomical, and Growth Deficits in Fetal Alcohol Spectrum Disorders

**DOI:** 10.1371/journal.pone.0047499

**Published:** 2012-10-19

**Authors:** Echoleah S. Rufer, Tuan D. Tran, Megan M. Attridge, Matthew E. Andrzejewski, George R. Flentke, Susan M. Smith

**Affiliations:** 1 Molecular and Environmental Toxicology Center, University of Wisconsin-Madison, Madison, Wisconsin, United States of America; 2 Department of Nutritional Sciences, University of Wisconsin-Madison, Madison, Wisconsin, United States of America; 3 Waisman Center, University of Wisconsin-Madison, Madison, Wisconsin, United States of America; 4 Multidisciplinary Studies Program in Neuroscience, Department of Psychology, East Carolina University, Greenville, North Carolina, United States of America; Pomona College, United States of America

## Abstract

Fetal alcohol spectrum disorders (FASD) are the leading non-genetic cause of neurodevelopmental disability in children. Although alcohol is clearly teratogenic, environmental factors such as gravidity and socioeconomic status significantly modify individual FASD risk despite equivalent alcohol intake. An explanation for this variability could inform FASD prevention. Here we show that the most common nutritional deficiency of pregnancy, iron deficiency without anemia (ID), is a potent and synergistic modifier of FASD risk. Using an established rat model of third trimester-equivalent binge drinking, we show that ID significantly interacts with alcohol to impair postnatal somatic growth, associative learning, and white matter formation, as compared with either insult separately. For the associative learning and myelination deficits, the ID-alcohol interaction was synergistic and the deficits persisted even after the offsprings’ iron status had normalized. Importantly, the observed deficits in the ID-alcohol animals comprise key diagnostic criteria of FASD. Other neurobehaviors were normal, showing the ID-alcohol interaction was selective and did not reflect a generalized malnutrition. Importantly ID worsened FASD outcome even though the mothers lacked overt anemia; thus diagnostics that emphasize hematological markers will not identify pregnancies at-risk. This is the first direct demonstration that, as suggested by clinical studies, maternal iron status has a unique influence upon FASD outcome. While alcohol is unquestionably teratogenic, this ID-alcohol interaction likely represents a significant portion of FASD diagnoses because ID is more common in alcohol-abusing pregnancies than generally appreciated. Iron status may also underlie the associations between FASD and parity or socioeconomic status. We propose that increased attention to normalizing maternal iron status will substantially improve FASD outcome, even if maternal alcohol abuse continues. These findings offer novel insights into how alcohol damages the developing brain.

## Introduction

Alcohol abuse is a major global health concern. Among its devastating consequences is Fetal Alcohol Spectrum Disorders (FASD). FASD is the greatest non-genetic cause of neurodevelopmental disability in children, affecting 9.1–50 per 1000 live births and 68.0–89.2 per 1000 in populations where alcohol abuse is common [1]–[3]. Exposure of the developing brain to alcohol causes permanent neurological damage and a distinctive behavioral profile that affects learning, memory, attention, executive functions, and motor skills [4], [5]. FASD prevention is challenging because of social stigmas surrounding alcohol abuse, the limited use of perinatal alcohol screening, and the failure of many alcoholics to admit their drinking behavior. Thus many at-risk pregnancies are never identified for intervention. Consequently there is a high priority for treatments that ameliorate alcohol’s neurotoxicity and especially gestational interventions that do not require knowledge of alcohol abuse [6].

An additional complication in FASD prevention is that the severity of alcohol’s effects can vary widely even after controlling for known modifying factors including pattern and quantity of alcohol intake and genetic variation in alcohol clearance kinetics. This variability suggests that factors in addition to alcohol contribute to the FASD phenotype. Identification of these modifiers could significantly inform FASD prevention. An important insight has emerged from the identification of several factors that modify alcohol’s effect: 1) increased maternal parity and maternal age, which increase FASD severity and frequency, and 2) higher maternal socioeconomic status (SES), which decreases FASD severity and frequency [3], [7]–[9]. We hypothesize that these associations may, in part, reflect their roles as surrogate markers for conditions that modify alcohol’s neurotoxicity. One likely condition is maternal nutritional status, as it is significantly influenced by SES, gravidity, and maternal age.

In considering the nutritional inadequacies experienced in alcoholic women and pregnancy, we selected iron for examination. Gestational ID is a significant public health concern. In the U.S., iron deficiency (ID) affects 22% of women aged 12–49 years and 7% of toddlers with higher rates in developing countries [10]. Increased gravidity and parity substantially reduce maternal iron status because the developing fetus and increased maternal vasculature draw significantly from maternal iron reserves. To address this, pregnant women are often prescribed iron supplements but compliance is poor due to adverse side effects such as constipation. Consequently, ID is the most common nutritional deficiency in women of child-bearing age. In the offspring it causes cognitive and behavioral deficits that involve many of the same domains affected in FASD including learning, attention, executive function, and motor skills [11], [12]. These deficits persist even after iron status is normalized and reflect iron’s roles during brain development for neurotransmitter metabolism and myelination as well as oxidative phosphorylation. Importantly, these behavioral deficits occur in the offspring even when overt maternal anemia is absent [12], [13]. This is because, when maternal iron is limiting, her stores are insufficient to meet both her needs and those of her rapidly growing offspring [13]. Thus, maternal iron status can significantly understate the magnitude of ID experienced by the offspring.

We hypothesized that severe aspects of FASD result from an interaction between alcohol and poor maternal nutrient status. It is poorly understood how nutritional status affects the offspring’s vulnerability to alcohol’s neurodevelopmental damage. Several clinical studies note an association between low iron status and FASD risk. In Cape Coloured children of South Africa with fetal alcohol syndrome (FAS), those children having the slowest growth trajectories - a hallmark of FAS - had the highest prevalence of ID anemia [14]. In an otherwise well-nourished cohort of pregnant women in the U.S., those with the highest alcohol consumption had a high risk for ID-anemia [15]. Additionally, prenatal alcohol exposure was shown to reduce brain iron content and alter iron homeostasis in the rat [16]. Here we use a rat model to directly test the hypothesis that maternal iron status significantly influences FASD outcome. We modeled the most common ID condition in women, where liver iron stores are largely depleted but hematological indicators are largely normal, such that the mother is not overtly anemic and thus the deficiency might not be diagnosed or treated aggressively [17]. The offspring received binge alcohol exposure during the third trimester-equivalent, the brain growth spurt period, which is sensitive to alcohol’s neurotoxicity [18]. We evaluated key outcomes of FASD including body growth, brain dysmorphology, and neurobehavioral outcomes. We hypothesized that alcohol’s effects on these diagnostic endpoints are partly attributed to, and exacerbated by, a synergistic interaction between alcohol and poor iron status.

## Results

### Iron and Alcohol Status

**Figure 1:**
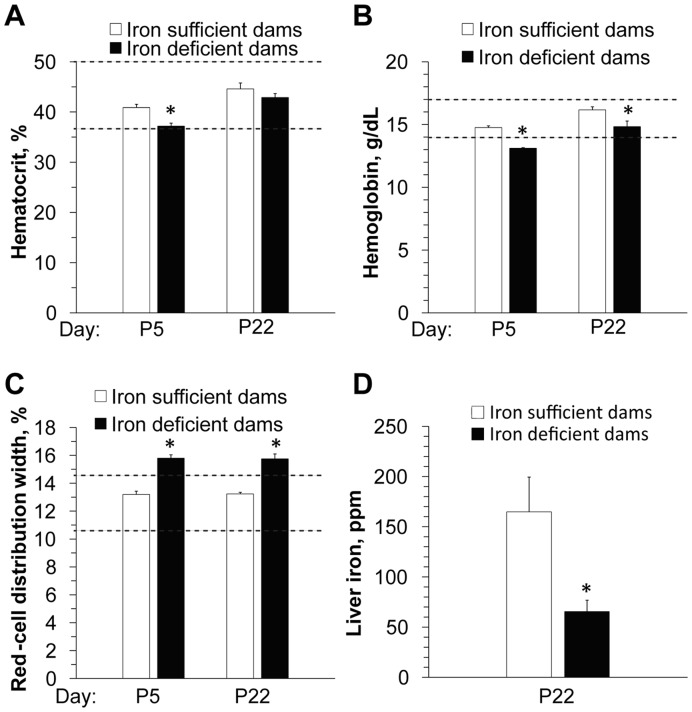
Rat dams fed an ID diet have moderate ID without anemia. Hematocrit (**A**), hemoglobin (**B**), red-cell distribution width (**C**), and liver iron (**D**) in rat dams on P5 and/or P22 fed IS or ID diets. Dashed lines indicate the normal reference range for non-pregnant adult rats [19]. N = 6–8 rats per group at each time point. *, significantly different from IS rats at the same time point as determined by linear mixed modeling.

**Figure 2:**
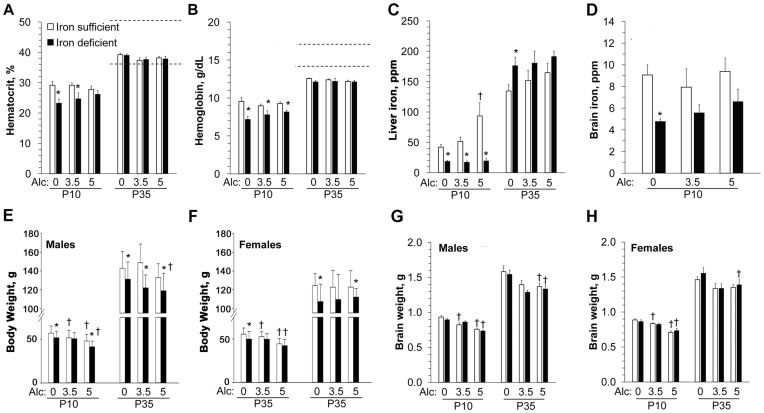
Reduced iron status and body growth in offspring of ID dams. (**A–D**) ID offspring are anemic at P10 but iron repletion normalizes their iron status by P35. Hematocrit (**A**), hemoglobin (**B**), liver iron (**C**), and brain iron (**D**) in IS or ID offspring at P10 and/or P35. Dashed lines indicate the normal reference range for non-pregnant adults and do not fully apply to growing animals [19]. N = 5–13 rats per treatment group at each time point. (**E-F**) Effect of maternal ID and postnatal alcohol on body weight of P10 and P35 male (**E**) and female (**F**) pups treated with indicated alcohol dose. N≥22 rats per treatment group per sex. (**G–H**) Alcohol significantly diminished male (**G**) and female (**H**) whole brain weight on P10 and P35 which was not further altered by iron status. N≥5 rats per treatment group per sex. *, significantly different from age-matched IS pups receiving the same Alcohol dose; **†**, significantly different from age-matched animals receiving 0 g/kg alcohol within the same iron status.

Iron status indicators were all within the normal range for the Iron-Sufficient (IS) dams at postnatal day 5 (P5; Figure 1, Table S1) [19]. The iron status indicators for the P5 ID dams were also normal (Figure 1, Table S1) except for modest changes in hemoglobin (decreased 11%) and red-cell distribution width (increased 20%) from normative values; these values had largely normalized by P22. Iron status did not affect litter size (IS 9.5±3.3; ID 9.9±2.7) and pup survival. In contrast, the ID offspring were anemic at P10 as evidenced by decreased hematocrit (F_(1,18) = _16.0, *P* = 0.001), hemoglobin (F_(1,14) = _18.0, *P*<0.001), and liver iron (F_(1,13) = _18.3, *P* = 0.001; Figure 2A–C, Table S2), and their brain iron content was significantly decreased (F_(1,19) = _8.6, *P* = 0.008; Figure 2D). Their poorer iron status reflected the inability of maternal ID to meet the offspring’s iron needs [13], [20]. By P35 offspring iron status was normal and alcohol did not further alter their iron indicators (Figure 2A–C, Table S2). IS offspring receiving 5 g/kg alcohol had elevated liver iron at P10 but not P35 (Figure 2C), and this was likely caused by alcohol’s known repression of hepcidin, which negatively regulates intestinal iron absorption [21]. Iron status did not affect the peak blood alcohol levels or alcohol clearance kinetics in the offspring (Figure S1).

### Growth Outcomes

**Figure 3:**
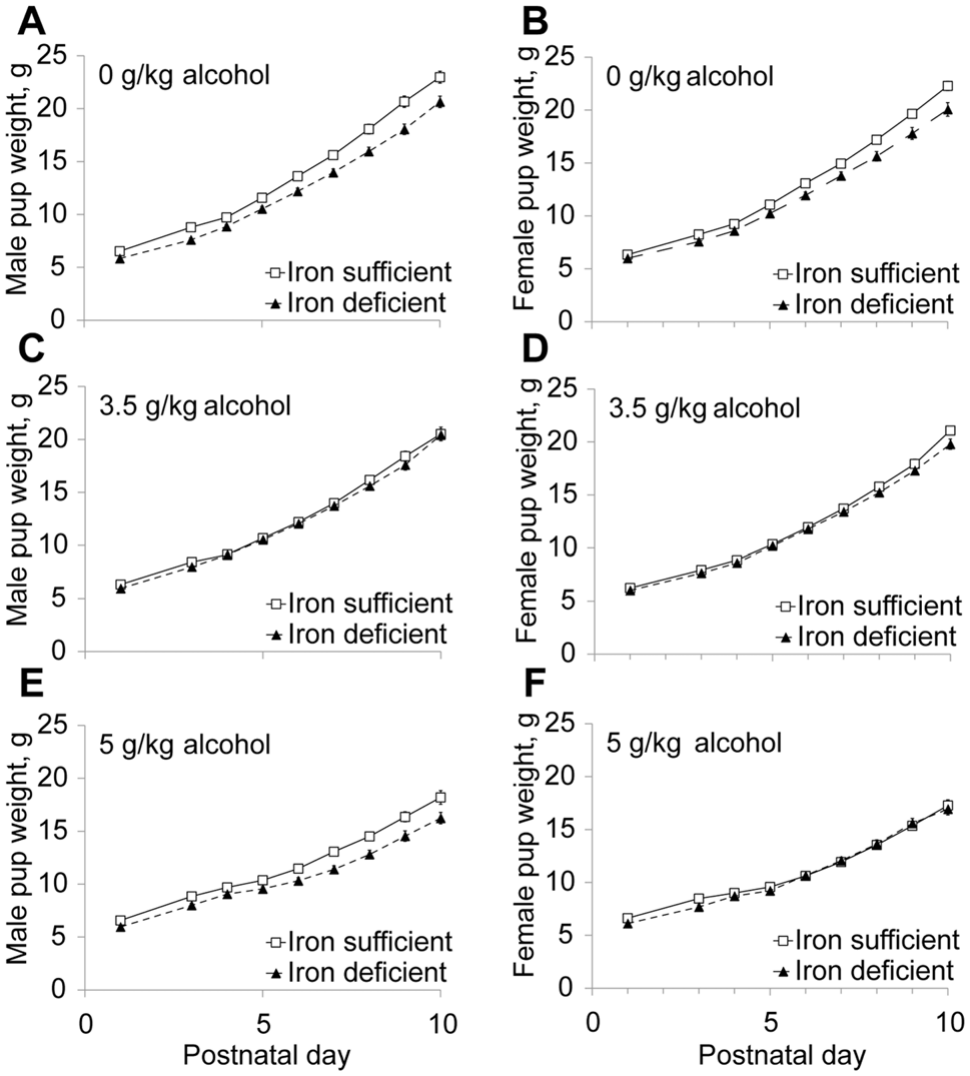
Maternal ID and alcohol interact to adversely affect somatic growth in males but not females. Body weight on P1–10 in male (A, C, E) and female (B, D, F) pups treated with 0 (A, B), 3.5 (C, D), or 5 g/kg alcohol (E, F). N≥22 rats per treatment group per sex. ID-only reduced growth in both males and females. Alcohol-only did not affect growth in either sex, but interacted with ID to reduce growth in males and not females. Markers of significance were omitted for clarity purposes.

Growth retardation is a key diagnostic criterion in FAS [4]. We found a complex interaction between Alcohol and ID that reduced postnatal body growth (*P*<0.001; Figure 2E–F, Figure 3) and this interaction was additionally influenced by sex (Age×Iron status×Alcohol×Sex interaction: F_(99,6533) = _1.75, *P*<0.001). In females, perinatal ID (F_(1,223)_ = 18.17, *P* = 0.000), but not alcohol, significantly reduced body weight across the study period (P1–P35) and the ID-alcohol combination did not further reduce their growth (Figure 3). For males, ID but not alcohol similarly reduced overall body growth from P1 to P35 (F_(1,217)_ = 18.11, *P* = 0.000) but, in contrast with their female littermates, there was a significant ID-alcohol interaction that further reduced the male offsprings’ growth (F_(2,215)_ = 22.66, *P* = 0.000). Separate ANOVAs were conducted at key developmental ages to help elucidate the Age×Iron Status×Alcohol×Sex interaction. At P10, alcohol significantly reduced body weight in both IS and ID males (Alcohol: F_(2,161)_ = 27.72, *P* = 0.000; Iron Status: F_(1,162)_ = 9.44, *P* = 0.002) and in IS but not ID females (Alcohol: F_(2,169)_ = 32.46, *P* = 0.000; Iron Status: F_(1,170)_ = 8.46, *P* = 0.004). By P35, body weights were still reduced by perinatal ID (females: F_(1,68) = _10.52, *P* = 0.002; males: F_(1,71) = _19.24, *P* = 0.000) and alcohol (males only: F_(1,71) = _3.33, *P* = 0.042), but an ID-alcohol interaction was no longer observed, suggesting that the affected offspring experienced catch-up growth. Brain weight was significantly decreased by alcohol, but not ID, in both sexes evaluated at P10 (*F*_(2,138) = _85.9, *P*<0.001) and at P35 (*F*_(2,92)_ = 19.3, *P*<0.001) (Figure 2G–H). These data suggest that iron status is an important modifier of somatic growth in males with FAS.

### Neurobehavioral Outcomes

**Figure 4:**
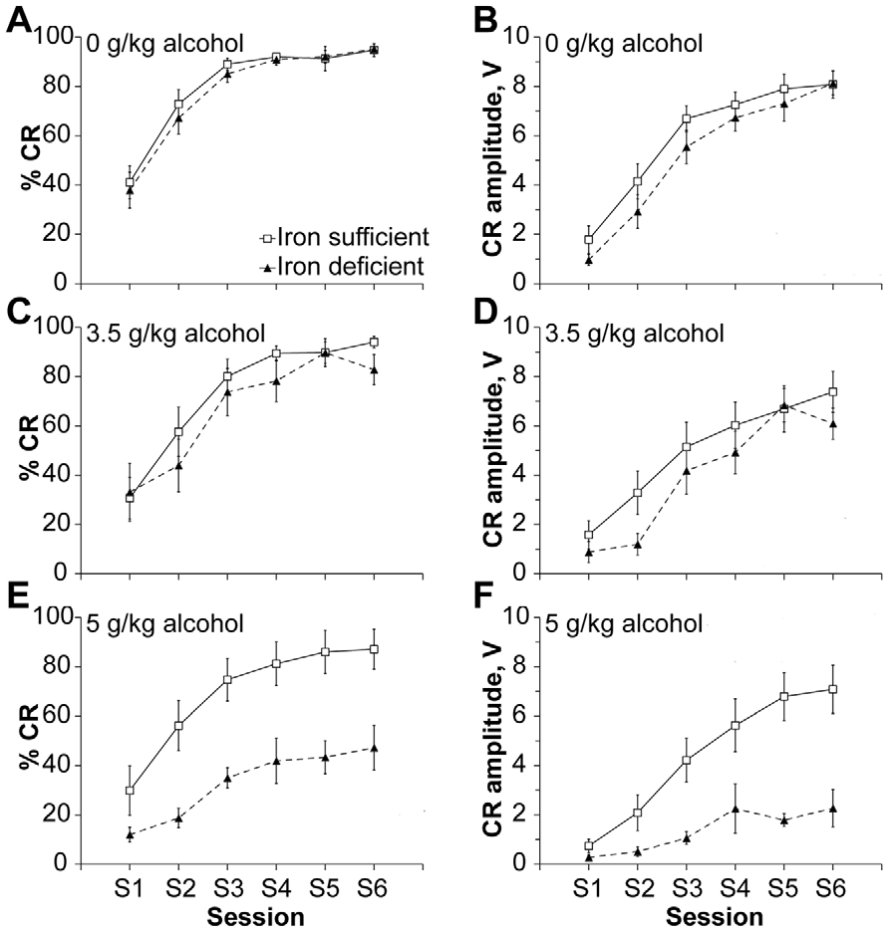
Maternal IDAA profoundly exacerbates alcohol-induced deficits in offspring’s delay ECC performance. Percent acquisition and amplitude of conditioned responses (CR) in IS and ID P35 offspring receiving 0 (**A, B**), 3.5 (**C, D**), or 5 (**E, F**) g/kg alcohol per day during the brain growth spurt. N = 9–16 rats per treatment group. There were significant main effects of Iron status, Alcohol dose, and an Iron status×Alcohol dose interaction. The interactive effect of Iron status and Alcohol was observed in ID rats that received 5 g/kg alcohol (**E, F**), where they were more impaired in acquiring CRs compared to IS rats that received 5 g/kg alcohol. Markers of significance omitted for clarity purposes.

Neurobehavioral disabilities are the most devastating clinical outcomes in FASD. We evaluated a range of these after iron repletion to identify those modulated by developmental ID. Behavioral testing was done at adolescence (P32–40) when the iron status indicators of the ID offspring had normalized (Figure 2). Short-delay eyeblink classical conditioning (ECC) is a cerebellum-dependent associative learning task and its impairment may have high diagnostic sensitivity for FASD [22]. For brevity, results for individual sexes are not reported because no significant sex differences were observed within any particular level of a factor (alcohol or iron diet), and when appropriate, analyses consist of data combined for both sexes. Separately, alcohol and ID did not alter ECC conditioned response (CR) acquisition or amplitude (Figure 4), as expected from the unconditioned stimulus intensity used here [23]. In contrast, the alcohol-ID combination was profoundly synergistic and substantially impaired ECC learning. CRs of ID animals receiving 5 g/kg alcohol were only 30% of the IS asymptote for frequency (*P* = 0.037) and 35% of the IS asymptote for amplitude (*P* = 0.006; Figure 4E, F). There was a significant interaction between Iron status×Alcohol dose (F_Frequency(2,55)_ = 4.1, *P* = 0.022; F_Amplitude(2,55)_ = 3.32, *P* = 0.042). ID animals showed a significant main effect of Alcohol on CR frequency (F_(2,23)_ = 17.33, *P*<0.005) and amplitude (F_(2,23)_ = 17.75, *P*<0.005) and ID animals receiving 5 g/kg had significantly impaired CR frequency and amplitude compared to the 0 (*P*_Frequency, Amplitude_ <0.005) and 3.5 g/kg groups (*P*_Frequency_ = 0.001; *P*_Amplitude_ = 0.001).

Although there was some degree of learning across training sessions regardless of alcohol dose or iron status, the ID +5 g/kg alcohol group reached a significantly lower asymptote (mean of sessions 5 and 6) compared to the IS +5 g/kg alcohol group (F_Frequency(1,17)_ = 6.98, *P* = 0.017; F_Amplitude(1,17)_ = 13.82, *P* = 0.002). There was a significant Iron status×Alcohol×Session interaction for CR amplitude (F_(10,275)_ = 3.67, *P*<0.005) but not frequency. The impairments could not be explained by an effect of alcohol or iron status upon sensory responding (Figure S2). The results suggested that ID profoundly exacerbated alcohol’s damage to associative learning.

**Figure 5:**
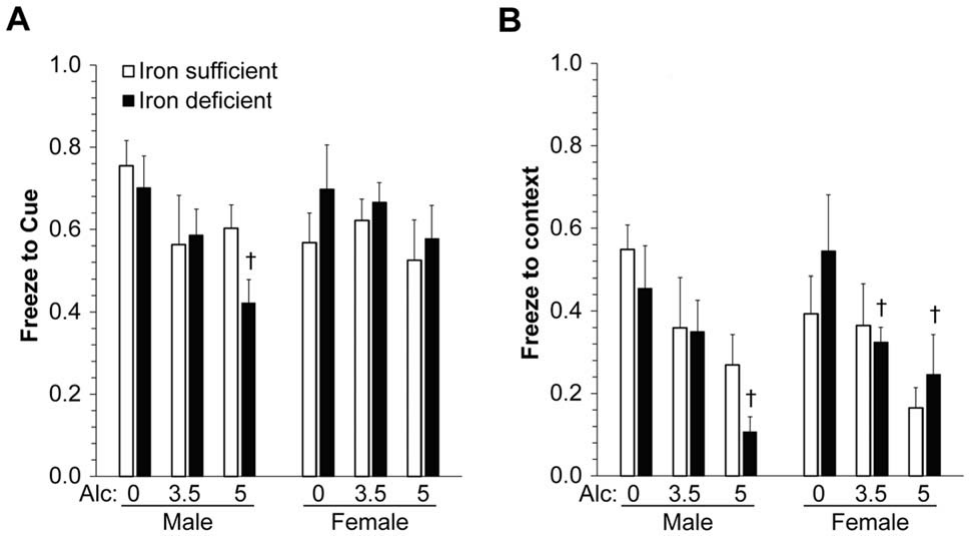
Maternal ID modulates cued and contextual fear conditioning in alcohol-exposed pups. Percent freezing to cue (**A**) and context (**B**) in male and female pups. N = 7–11 rats per treatment group per sex. There was a main effect of Alcohol within ID males on cued (F_(2,16) = _6.1, *P* = 0.011) and within ID males (F_(2,6) = _7.4, *P* = 0.024) and females (F_(2,14) = _6.9, *P* = 0.008) on contextual fear conditioning. **†**, significantly different from 0 g/kg alcohol within the same Iron status.

The alcohol-ID interaction extended to other forms of associative learning, using auditory-cued (amygdala-dependent) and contextual (amygdala- and hippocampus-dependent) fear conditioning. Although there was no significant main effect of Sex, data are presented by Sex as there was an apparent differential response in males and females. Cue-induced CR was significantly impaired by alcohol in ID males (F_(2,16) = _6.14, *P = *0.011; Figure 5A), but not in ID females or in IS animals of either sex. For contextual CR, there was a significant main effect of Alcohol in ID males (F_(2,6) = _7.38, *P = *0.024; Figure 5B) and females (F_(2,14) = _6.95, *P* = 0.008), but not in IS males (F_(2,17) = _2.03, *P = *0.161) or females (F_(2,11) = _1.40, *P = *0.288). At 5 g/kg alcohol, ID males had less freezing in comparison to IS males (10.7±3.7% vs. 26.9±7.4%, respectively) but this was not significant. In summary, ID exacerbated alcohol-induced deficits in several forms of associative learning.

In contrast, several other tasks did not show an alcohol-ID interaction (Figure S3). Forelimb grip strength was unchanged, consistent with the ID animals’ now-normalized iron status and suggesting that muscle weakness was not a factor in other behavioral deficits. Motor coordination tasks (parallel bar traversal, rope climb, gait) were also unaffected except for alcohol’s impairment of parallel bar traversal (F_(2,49)_ = 5.129, *P* = 0.009). Thus the ID-alcohol interaction selectively targeted a subset of alcohol-dependent behavioral deficits.

### Neuroanatomical Outcomes

**Figure 6:**
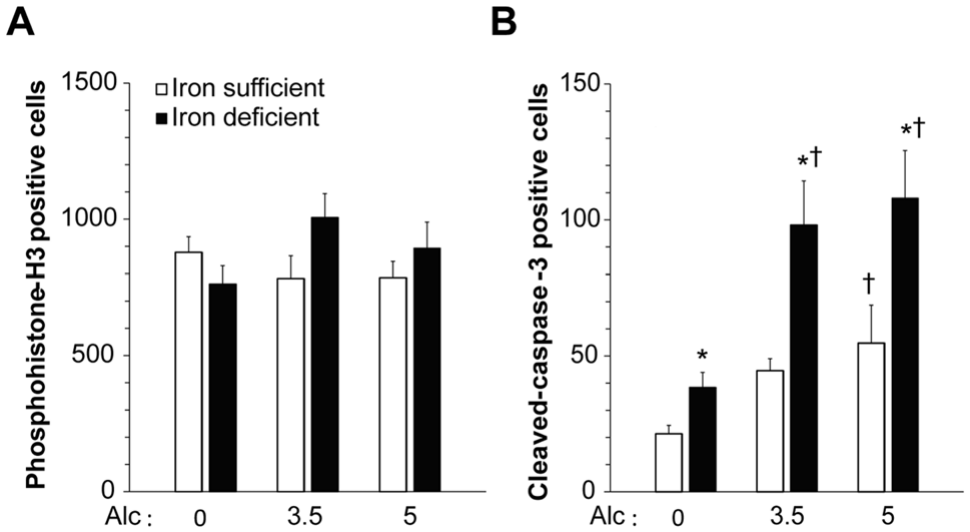
Maternal ID increases alcohol-induced cerebellar apoptosis at P10. (**A**) There were no significant main effects of Iron status or Alcohol dose on proliferation, quantified using phosphorylated histone-H3 immunoreactivity. (**B**) Apoptosis, assessed using cleaved-caspase-3 immunoreactivity, was decreased in the P10 cerebellum. Both ID (*P* = 004) and alcohol (*P* = 0.001) increased apoptosis and their effects were additive. N≥5 rats per treatment group. *, significantly different from IS pups within the same alcohol dose; **†**, significantly different from 0 g/kg alcohol within the same Iron status.

While ID itself impairs learning [11], [12], the learning deficits in the ID-alcohol animals could not be attributed to an acute nutritional insufficiency because they had consumed an IS diet since weaning and had a normalized iron status when tested at adolescence (Table S2). This suggested a long-term impact of the alcohol-ID interaction upon neurodevelopment. Neuroanatomical changes in FASD include increased neurodegeneration, altered cellularity, and impaired white matter formation [18], [24]–[28]. We evaluated the cerebellum because the profoundly impaired ECC associative learning observed here depends on a well-documented neurocircuitry within that structure [29]. Examination of the ID-alcohol cerebellum revealed significant microstructural abnormalities. At P10 the overall cerebellar morphology appeared normal (Figure S4). The number of proliferating cells within the P10 cerebellum was unaffected by either the alcohol or ID treatment (Figure 6A). In contrast, alcohol-alone (5.0 g/kg) and ID-alone increased the apoptosis level in P10 cerebellum, and cell death was further increased by the ID-alcohol combination (Figure 6B). ID doubled the incidence of neuronal apoptosis as compared to IS pups at the same alcohol dose (*F*_3.5 g/kg (1,10) = _13.7, *P = *0.004 and *F*_5 g/kg (1,11) = _5.38, *P = *0.041). This effect was likely additive as there was no Iron status×Alcohol interaction. The apoptotic cells were evenly distributed across the cerebellar lobules and cell types in all treatment groups. Thus, while alcohol exposure caused significant apoptotic neurodegeneration within the cerebellum, an insufficiency of brain iron content substantially exaggerated those losses. This suggested that neurodegenerative losses within the cerebellum contributed to the learning deficits of the ID-alcohol offspring.

**Figure 7:**
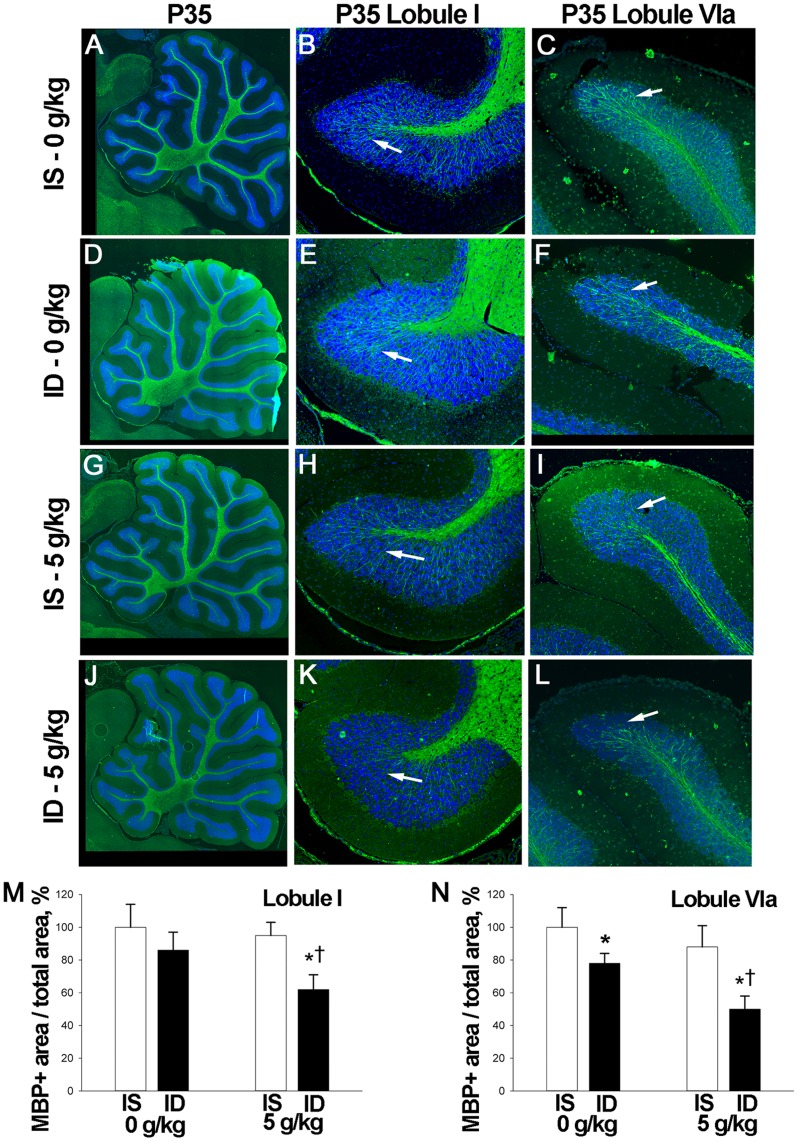
ID-alcohol exposure impairs myelination. **A–L,** Immunostain for myelin basic protein (MBP) of P35 cerebellum following the indicated treatments. **B, E, H, K** are the corresponding enlargements of Lobule I, and **C, F, I, L** are enlargements of Lobule VIa. There were fewer myelin tracts within the granule cell layer (arrows) in ID-alcohol cerebellum (**K, L** ) compared with controls (**B, C**) or alcohol-only (**H, I** ) or ID (**E, F**), and confirmed by quantifying the MBP^+^ area within the granule cell layer for lobule I (**M**) and lobule VIa (**N**). N = 6–8 rats per group. * Significantly different from IS pups at same alcohol dose, **†** significantly different from 0 g/kg alcohol within same iron status.

Both prenatal alcohol and gestational ID adversely affect myelination and cause structural changes in the white matter of multiple brain regions including the cerebellum [25]–[28], [30], [31]. We evaluated this at P35 by quantifying the distribution of myelin basic protein (MBP) within the granule cell layer (GCL) for two distinct cerebellar regions, Lobule I and Lobule VIa. We found that, separately, alcohol and ID did not affect the myelination content within the GCL of Lobule I(Figure 7), and within Lobule VIa GCL, ID-only but not alcohol-only produced a modest decrease in myelination. In contrast, there was a significant interaction between alcohol and iron status to reduce the myelin content within the GCL of both Lobule I (Figure 7I; F_(1,21)_ = 4.947, *P* = 0.037) and Lobule VIa (Figure 7J; F_(1,24)_ = 5.164, *P* = 0.032). For both lobules, myelin comprised a smaller percentage of the GCL as compared with alcohol or ID treatment individually. These data suggest that the white matter deficits seen in FASD might be partly attributed to poor maternal iron status during the alcohol exposure period.

## Discussion

Several studies have suggested that iron status may have a unique, modifying influence upon the clinical outcomes in FASD and this work provides the first direct demonstration for this hypothesis. We have demonstrated that maternal nutritional status, and specifically maternal iron insufficiency, is an important and powerful modifier of neurobehavior and growth in this rat model of FASD. The alcohol-exposed offspring born to mothers with low iron reserves had significant reductions in somatic growth, associative learning, myelination, and neuronal survival. In contrast, when maternal iron status was adequate, alcohol’s neurodevelopmental damage was mitigated and comparatively modest deficits in learning, body growth, and neuroanatomy were found. For several measures the impact of alcohol and ID was synergistic and not additive. Importantly, the deficits in the ID-alcohol animals represent part of the diagnostic criteria of Fetal Alcohol Syndrome, which represents the severe end of the FASD diagnostic spectrum [4], [5]. These characteristics include reduced somatic growth trajectories, enhanced neuronal and myelin losses, and deficits in associative learning. It is indisputable that alcohol is a potent neurodevelopmental teratogen and these data show that comorbid conditions can significantly influence the final outcome. Although there is a long-standing assumption that maternal nutritional status has a significant contribution to FASD [32]–[34], this hypothesis has not been systematically analyzed. Alcohol abuse is associated with higher risk for several nutritional deficiencies including iron, zinc, copper, thiamin, vitamin A, and perhaps choline; alcohol may also alter nutrient metabolism and requirements. More work is needed to identify additional nutritional factors that could affect FASD outcome. However, because ID is the most common nutritional deficiency in women of child-bearing age [10], an ID-alcohol interaction likely represents a significant portion of those receiving a FASD or FAS diagnosis. We propose that the treatment of maternal iron inadequacy and normalization of iron status will substantially attenuate the damage caused by prenatal alcohol exposure in a significant percentage of children at risk for FASD.

These findings have significant public health implications given the high rates of both alcohol abuse and ID in childbearing-age women of both industrialized and less-developed nations [1]–[3], [10]. For male and post-menopausal female alcoholics, iron overload is common due to alcohol’s dysregulation of hepcidin and subsequent enhancement of liver iron stores [21], [35]. However, the situation differs for women of child-bearing age due to their regular iron losses from menses and pregnancy; moreover, alcohol’s ability to enhance dietary iron absorption is irrelevant if the diet is iron-inadequate. Iron deficiency is more common in alcohol-abusing pregnancies than currently appreciated [14], [15]. In an alcohol-abusing U.S. cohort [15], the pregnant women with the highest drinking rates (>8 drinks/day) had the poorest iron status and 75% (9/12) of those women had iron-deficient anemia. In a South African FASD cohort 72% of children born to binge-drinking women had both iron-deficient anemia (42% in non-drinkers) and the most pronounced growth retardation, a hallmark of FAS [14]. In addition to overt anemia, our data show that ID without anemia also poses a significant risk for adverse outcome. As others have shown and our own work confirms, when iron is limiting the mother cannot supply sufficient iron to meet the growing offspring’s needs. Thus the child has a greater risk for ID, anemia and low iron reserves as compared to its mother [13], [20]. In monitoring iron status, hematological indicators change slowly relative to iron stores and can significantly overestimate them, failing to identify those pregnancies with poor iron status and increased ID risk. For this reason, we endorse the adoption of more sensitive indicators of iron status such as the ratio of zinc protoporphyrin to heme, as these identify at-risk pregnancies more accurately than do hemoglobin, serum ferritin, or transferrin saturation, which are affected by factors other than iron stores [36]–[38]. Increased attention to iron status during pregnancy is a novel and likely successful strategy to address the significant public health problem of FASD and FAS.

Our data may also explain recent controversial findings that children born to light-drinking mothers have lower FASD risk compared with abstainers or high drinkers [39], [40]. This light-drinking group also had higher income and education compared with the other cohorts. The prevalence of gestational ID decreases as income and education rise, partly because of increased iron supplement use [41]–[43]. Thus light-drinking is likely associated with reduced risk for gestational ID. Similarly, our findings also inform the significant, positive associations between FAS and parity. A term pregnancy requires 1040 mg iron [44]. When iron intake is limiting, it is difficult to rebuild iron reserves during the interpregnancy interval and thus multiparous women have increased ID risk [41], [42]. Shorter interpregnancy intervals are associated with low income and high parity [45]. We suggest that maternal iron reserves and iron supplement use, and the environmental factors that modify these, partly explain the impact of parity, age, and socioeconomic status upon FASD risk.

How do alcohol and ID interact to produce the neurodevelopmental deficits seen here? This likely involves several mechanisms as both alcohol and ID have multiple effects upon the developing brain. These include reduced mitochondrial energy generation, reduced neuronal survival and synaptogenesis, and altered neurotransmitter activity [11], [13], [18], [24]. We show here that an important mechanism is a significant interaction between ID-alcohol to reduce myelination. There is growing evidence that white matter deficits including myelination problems are a significant hallmark of FASD. Diffusion tensor imaging of individuals with FASD reveals white matter deficits in multiple brain regions including the cerebellum, corpus callosum, brainstem, temporal lobe, and thalamus [25]–[28]. These changes correlate with specific cognitive impairments affecting executive function, math processing, visual-perception, visuomotor integration, and of special relevance to our findings, associative learning. The reduced MBP immunostaining seen in our alcohol-exposed animals supports both these clinical observations and animal FAS studies reporting reduced MBP, delayed myelination and altered axonogenesis [46]–[48]. The present work highlights the vulnerability of cerebellar myelination to alcohol’s damage during the third trimester equivalent. Iron also plays a prominent role in myelination; oligodendrocytes are iron-enriched and require iron for maturation into the myelin sheath [30], [31]. The significantly reduced myelination in our ID animals confirms that work and endorses the importance of perinatal iron adequacy for normal cerebellar development. Importantly, the addition of gestational ID during the alcohol exposure substantially exacerbates the myelination deficits as compared with either treatment individually. Thus maternal ID heightens the vulnerability of myelination to alcohol-mediated damage. While additional study is needed to ascertain if these MBP losses represent disrupted myelin formation, axon formation, or both, the present findings identify iron status as a significant contributor to the white matter deficits associated with FASD. Our data suggest that the most pronounced white matter losses in FASD may represent alcohol-exposed pregnancies in which iron deficiency was also present.

These myelination deficits likely contributed to the impaired associative learning of the ID-alcohol rats. Cerebellar white matter tracts, including those within Lobule VIa [49], [50], are essential participants in the delay ECC learning studied here and link Purkinje cells with granule cells in the cerebellar cortex, completing the circuit with the ocular musculature [29]. The substantial myelin losses seen within the Lobule VIa GCL of the ID-alcohol animals implies that the major afferent signals with cerebellar Purkinje cells involved in basic motor learning were compromised by the ID-alcohol combination. Such reductions could contribute to the poor learning of these animals in the delay ECC task. One caveat is that the overall timing of these animals’ CRs was not altered. However, the delay ECC learning task used here is an optimal ECC learning task and is acquired with relative ease compared with more difficult ECC tasks. Thus the lack of timing deficits may be the result of a myelin reduction threshold not being reached. Our conclusions are also supported by the recent clinical demonstration of similar, close associations between prenatal alcohol exposure, cerebellar white matter deficits, and poor learning performance using a trace ECC task similar to that studied here [28]. As that clinical population also experiences significant gestational ID as compared with non-alcohol-exposed controls within that community [14], the authors speculated that poor iron status may have heightened the offspring’s vulnerability to the adverse effects of prenatal alcohol exposure, perhaps through effects on myelination. Our data directly support this hypothesis and inform its mechanistic basis by showing a significant interaction between ID and alcohol to reduce cerebellar MBP content including regions that contribute to associative learning.

Children with FASD from this same community also show a strong association between poor iron status and reduced body growth [14], an association that was observed in this rat model. Gender has an additional modifying effect upon body weight in those with FASD, and pubertal males retain their smaller stature while females achieve normal weights through accumulation of fat mass [51]. A similar gender effect was seen here, and ID-alcohol interacted to reduce body growth in male but not female offspring. Males are more sensitive to ID than females due to their more rapid growth and increased muscle mass, both of which increase iron needs. The greater growth reductions in the ID-alcohol males vs. females may reflect this difference and endorses the conclusion by Carter et al. [14] that limiting iron stores may magnify the growth reductions of FASD, especially in males. Taken together, findings herein directly demonstrate that poor iron status heightens the offspring’s vulnerability to the growth, cognitive, and brain microstructural deficits caused by prenatal alcohol-exposure.

The critical period for the protective effects of iron repletion in the alcohol-exposed offspring was likely the alcohol exposure period itself, because the ID-alcohol offspring were iron-sufficient when the behavioral testing was performed at adolescence. Normalization of iron status post-weaning did not reverse their learning deficits. Under normal nutriture, the neonate receives most of its iron stores during the late third trimester and these stores cannot meet postnatal needs when maternal iron stores are limiting [11], [13]. Thus it is especially important to resolve iron deficiency prior to this critical period. Fortunately, there are proven, low-cost methods to enhance maternal and offspring iron status, including aggressive screening for women having low iron status but lacking overt anemia, the use of slow-release or low-dose iron supplements, delayed cord clamping at delivery, and increased breast-feeding [20], [43]. The significantly improved outcomes of the IS-alcohol over the ID-alcohol animals suggest that maternal iron supplements are accessible to the offspring despite the alcohol-exposure. This issue is important because clinical trials are underway to directly test the ability of nutritional supplements, including iron, to improve FASD outcomes [34]. However, an important caveat to such interventions is that alcohol abuse alters iron homeostasis in the adult [21], [35]. Perinatal alcohol exposure may similarly alter iron homeostasis and brain iron availability in the offspring [16], and studies are underway in our laboratory to examine this further. Although our understanding of how alcohol alters iron homeostasis is incomplete, our data support the importance of normalizing maternal iron status in alcohol-exposed pregnancies. In conclusion, our data demonstrate that aggressive screening and treatment of gestational ID in alcohol-using women, in the presence or absence of overt anemia, offers a safe and clinically achievable approach to substantially ameliorate the devastating consequences of FASD.

## Methods

### Animals

We used established models of alcohol exposure and ID [17], [18]. Nulliparous, 175 g Long-Evans female rats were fed an IS diet (TD.06016; Harlan-Teklad, Madison, WI; 100 ppm iron) to gestational day 5 (GD5). The morning a vaginal plug was detected was GD0. IS animals were fed that diet until weaning (P22). ID dams were fed a 20 ppm iron diet (TD.06013; Harlan-Teklad, Madison, WI) from GD5–GD13.5 and P7–P22 and 4 ppm iron diet (TD.80396) from GD13.5-P7 to maintain a moderate ID status. All pups were fed an iron adequate diet starting at weaning (ProLab RMH3000, PMI; 329 ppm iron). P4 litters were culled to 10 pups, striving for equal sex distribution. Protocols were approved by the Institutional Animal Care and Use Committees. Hematological measures included complete blood counts (UW Veterinary School) and serum transferrin saturation, iron, and total iron binding capacity (Cornell Univ. Animal Health Diagnostic Center). Tissue mineral content was quantified using ICP-OES (UW Soil Science Analysis Laboratory).

### Ethanol Exposure

Littermate pups received 0, 3.5 or 5.0 g ethanol/kg body weight in milk (Carnation nonfat dry milk +5% corn oil; 0.3 µg Fe/ml; 0.028 ml/g body weight) via gastric gavage, given as two half-doses 2 hr apart daily during the brain growth spurt (P4–9); normal rat milk contains 5.3*±*0.37**µg iron/ml [52]. This models episodic binge drinking in the third-trimester equivalent [18]. To control food intake during the dosing period, the dam was removed from the home cage until the alcohol pups recovered (∼4–6 hrs); thus at the 4 hr dosing all pups received a third, milk-only dose (four on P4) to sustain their nutriture. During this period, pups were kept warmed and in the home cage to minimize the potential stress of maternal separation. Blood alcohol content was measured in pups studied separately and quantified using Analox GM7 instrumentation (London, UK) according to the manufacturer’s protocol.

### Immunostaining

Midsagittal 7 µm paraffin sections of cerebellum were evaluated for proliferation (rabbit IgG polyclonal anti-phospho-histone-H3, 5A1, 1∶1,000; Upstate #06–570) and apoptosis (rabbit monoclonal IgG anti-cleaved-caspase-3, 5A1E, 1∶200; Cell Signaling Technologies #9664) followed by Alexa488-coupled secondary antibody (Molecular Probes). Nuclei were stained with DAPI. All immunopositive cells were counted by treatment-blinded observers in at least 2 serial sections per cerebellum and averaged.

Parallel sections were similarly stained for MBP (mouse IgG1 anti-MBP, SMI-94, 1∶1000; Abcam #24567) followed by the relevant Alexa488-conjugated donkey secondary antibody and DAPI counterstain. Slides were stained and processed as a single batch. Digital images were captured using identical exposure conditions, and the threshold gates to remove specular highlights and fluorescent background were applied uniformly. The GCL (DAPI^+^) layer of Lobules I and VIa was defined and the number of MBP^+^ pixels within the GCL was quantified using the analytical tools of Adobe Photoshop CS4. Results were expressed as the number of MBP(FITC)^+^ pixels within the GCL area.

### Short Delay Eyeblink Classical Conditioning

This was performed as described [53]. In brief, P30 rats were outfitted with an EMG “headstage” and ocular stimulating electrode. Animals were presented a 380 msec tone (CS, 2.8 kHz, 80 dB) that co-terminated with a 100 msec, 2.0 mA periorbital shock (US); the inter-stimulus interval was 280 msec. Animals received two training sessions daily on P32–34. Within-day sessions were 4 hr apart and consisted of 100 trials (90 paired CS-US, 10 CS-alone) with an average inter-trial interval of 30 sec (range = 18 to 42 sec). Data were pre-screened for acceptability using established criteria [53], [54]. Each trial epoch was analyzed as four discrete sampling periods: (1) a 280-msec pre-CS period that measured baseline activity, (2) a startle response (SR) period during the first 80 ms after CS onset, (3) a CR period measuring associative learning-related EMG activity (200 msec), and (4) a UR period measuring EMG activity after the onset of the US (140 msec). EMG activity exceeding the pre-CS baseline mean by at least 0.4 V (2 standard deviations) was registered as a SR, CR or UR during their respective sampling periods. Session means were obtained over 90 CS-US trials or 10 CS-alone trials. The relevant measures included frequency and amplitude of SRs, CRs and URs during paired CS-US trials.

### Open Field Activity

After a 30 min acclimation period, the P31 rat was centrally placed in a square arena (41×41×36 cm plexiglass divided into four equal squares) and videotaped for 10.5 minutes. After 30 sec of adaptation, horizontal locomotion (number of gridlines crossed with four paws) and vertical locomotion (rearing, defined as sustained posture with forepaws off the floor) were counted for 10 min from the recording. Total activity was defined as horizontal plus vertical locomotion.

### Tests of Motor Activity and Coordination

For *gait analysis* on P31, the rear paws were painted with non-toxic finger paint. Rats were placed on a paper within a ∼10 cm wide corridor and allowed to walk through. Lines were drawn from 4th and 5th paw prints and the angle formed was measured. *Parallel bar balance* on P32–34 tested the ability to successfully traverse the bars while confronting progressive increases in interrod width [55]. Immediately afterward, *rope climbing ability* was evaluated using successively thinner vertical ropes, where a score of 1 was an excellent performance with no prods and 5 signified the animal could not climb or hold rope [55]. *Forelimb grip strength* was evaluated at P35 using a grip strength meter (Columbus Instruments, Columbus, OH), recording the peak force before the grip release across 3 trials with a 10 sec rest period.

*Fear conditioning* was evaluated at P37 as described [56]. Rats were conditioned with two tone-shock pairings (30 sec, 5000 Hz, 90dB tone; 1 sec, 1 mA foot shock). For the contextual test, P38 rats were returned to the training chamber for 5 minutes (without the shock or auditory cue) and videotaped. Freezing time, defined as absence of all motion except breathing, was scored by a semi-blinded observer. Rats then were placed in a second chamber differing in appearance from the first and videotaped. At 2 and 4.5 minutes the same training tone was played for 30 sec and freeze time during the cue presentation was scored.

### Statistical Analysis

Data were subjected to linear mixed effect modeling followed by pairwise comparisons of the estimated marginal means with Sidak adjustments with PASW statistics 17.0. Litter was designated as a random factor and all others were fixed. For ECC data, all measures for paired CS-US trials (90 per session) were first analyzed using 2 (Sex)×2 (Iron status)×3 (Alcohol)×6 (Session) mixed ANOVAs, with Session as the repeated variable. Follow-up analyses involved reduced ANOVAs to confirm relevant comparisons being made. Significant main effects were analyzed using Tukey’s post hoc tests. Significant interactions were analyzed using simple effects analyses. *P*<0.05 was considered significant. All data are mean ± S.E.M.

## Supporting Information

Figure S1 **Iron status does not affect blood alcohol content (BAC).** BAC on P4 and P9 for pups gavaged with 3.5 (**A, B**) or 5.0 g/kg alcohol (**C, D**). Arrows indicate time of alcohol treatment (0 and 2 hours). Mean BAC ± SEM is shown. Each data point is the average of 2–4 pups.
(TIF)

Figure S2 **Startle responses in ECC testing.** (**A, B**) The mean percentage of startle responses (SR; pooled across 6 sessions of training) was <15% (range = 8–13–20%) and the mean SR amplitude was <1 V (range = 0.3–0.695 V). (**C, D**) Similarly, the pooled session means for unconditioned response (UR) frequency were >93%; measures averaged >96% and amplitudes ranged from 4.955–6.5 V for each Iron status×Alcohol group. These performance measures were not significantly different among groups and no interactive effects of Iron status×Alcohol dose were exhibited.
(TIF)

Figure S3 **Additional behavioral testing.** ID did not modulate alcohol’s effects on muscle strength, motor coordination, gait, or open field activity. Peak grip strength at P35 (**A**), percent successful parallel bar traversal on P32–34 (**B**), rope climb (**C**), gait angle (degrees; **D**), and total mobility (horizontal + vertical) in an open field on P31 (**E**).
(TIF)

Figure S4 **Cerebellar morphology.** Although alcohol-exposed pups (5 g/kg) had smaller cerebella, overt cerebellar morphology was normal in alcohol-treated offspring of IS and ID dams.
(TIF)

Table S1 Dam Iron Status. Blood and liver values at P5 and P22 for IS and ID dams used in this study.
(DOC)

Table S2 Pup Iron Status. Blood and liver values for IS and ID pups at P10 and P35 that were gavaged with 0, 3.5, or 5.0 g/kg alcohol daily from P4 to P9.
(DOC)
