# Supplementary figures and images for: Adequacy of Maternal Iron Status Protects against Behavioral, Neuroanatomical, and Growth Deficits in Fetal Alcohol Spectrum Disorders
Source: PLoS One. 2012 Oct 19;7(10):e47499. doi: 10.1371/journal.pone.0047499 (PMC3477151; doi:10.1371/journal.pone.0047499)

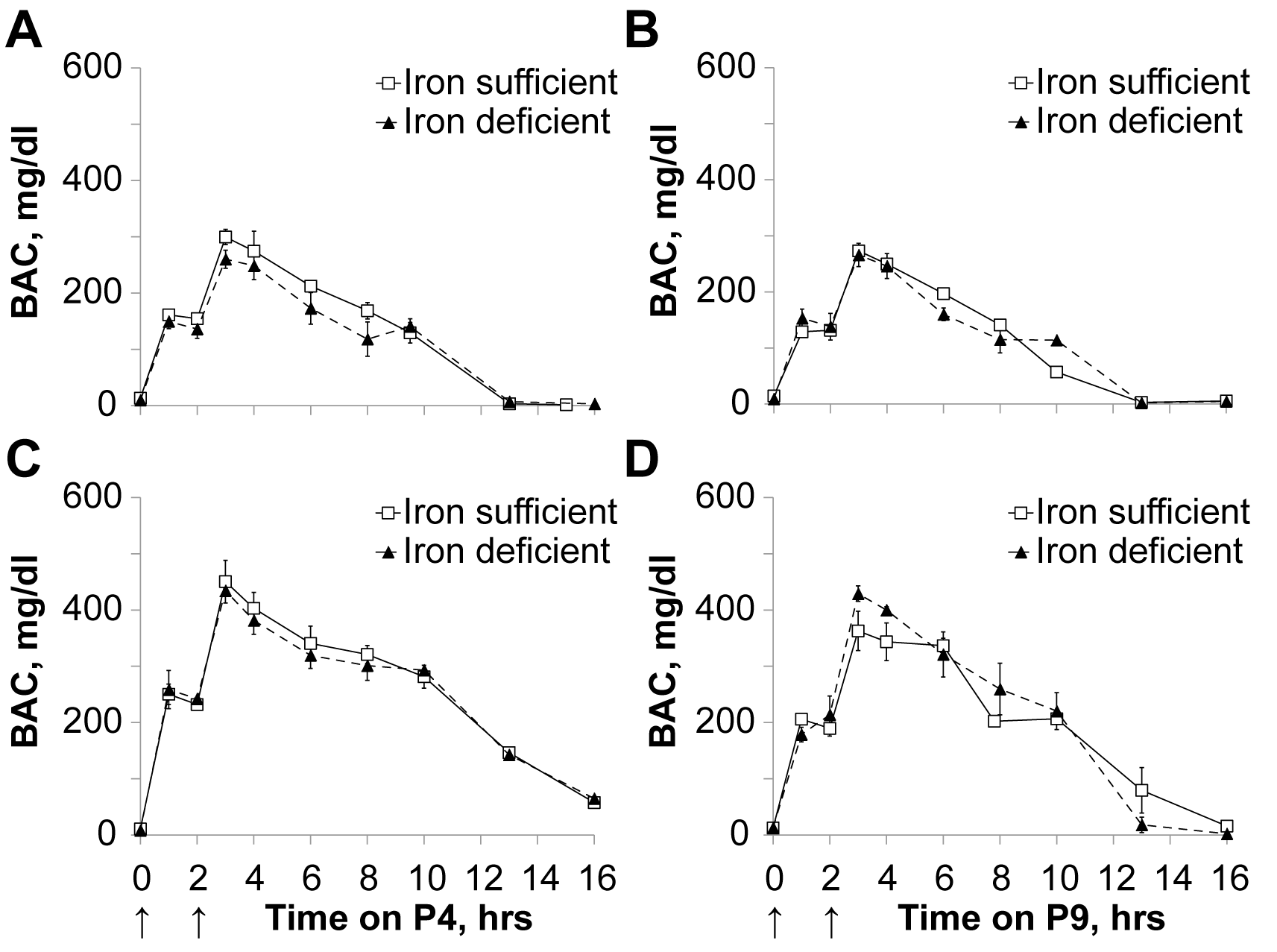

Supplement: Figure S1 — Iron status does not affect blood alcohol content (BAC). BAC on P4 and P9 for pups gavaged with 3.5 (A, B) or 5.0 g/kg alcohol (C, D). Arrows indicate time of alcohol treatment (0 and 2 hours). Mean BAC ± SEM is shown. Each data point is the average of 2–4 pups. (TIF) [file pone.0047499.s001.tif]

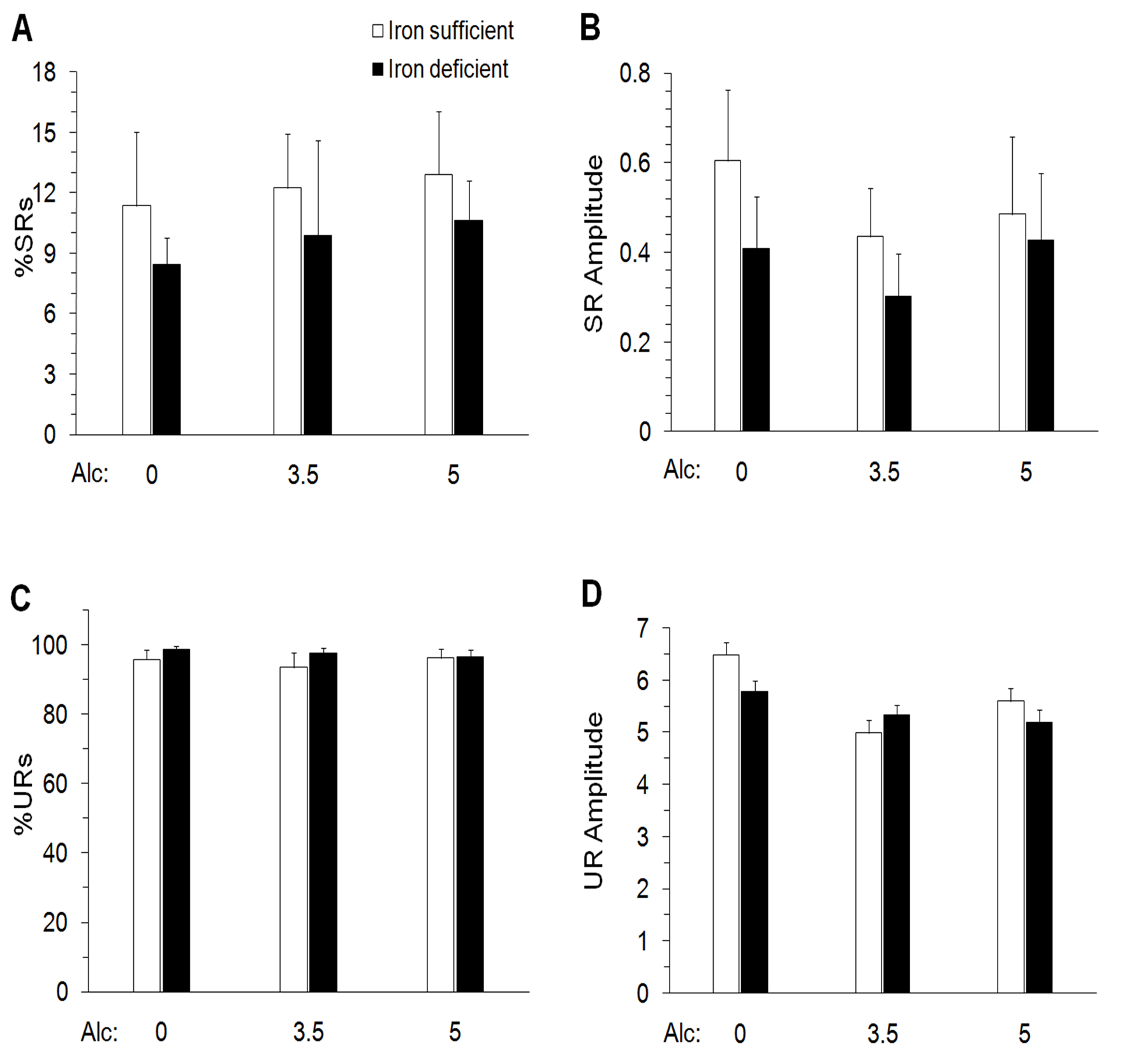

Supplement: Figure S2 — Startle responses in ECC testing. (A, B) The mean percentage of startle responses (SR; pooled across 6 sessions of training) was <15% (range = 8–13–20%) and the mean SR amplitude was <1 V (range = 0.3–0.695 V). (C, D) Similarly, the pooled session means for unconditioned response (UR) frequency were >93%; measures averaged >96% and amplitudes ranged from 4.955–6.5 V for each Iron status×Alcohol group. These performance measures were not significantly different among groups and no interactive effects of Iron status×Alcohol dose were exhibited. (TIF) [file pone.0047499.s002.tif]

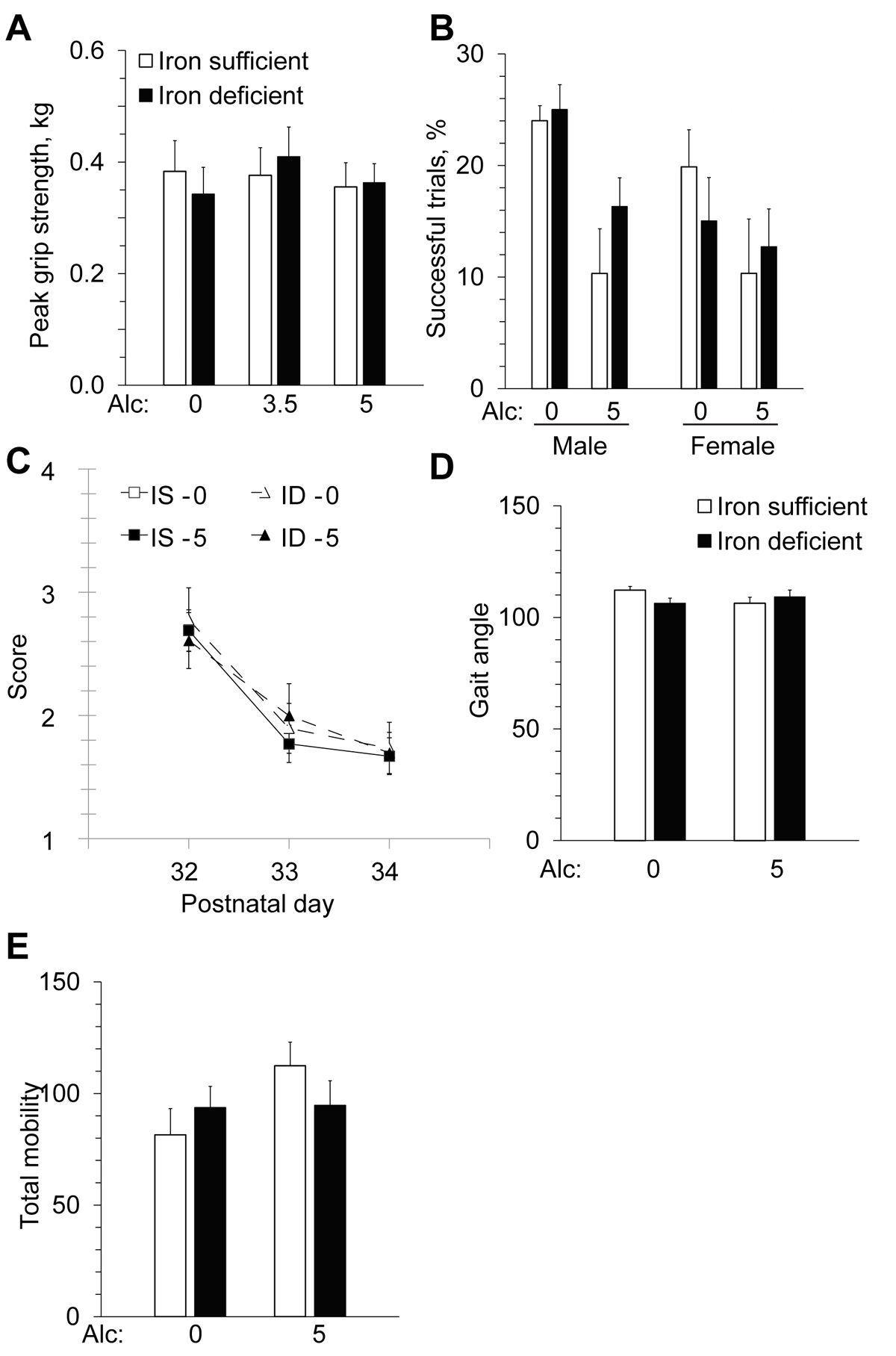

Supplement: Figure S3 — Additional behavioral testing. ID did not modulate alcohol’s effects on muscle strength, motor coordination, gait, or open field activity. Peak grip strength at P35 (A), percent successful parallel bar traversal on P32–34 (B), rope climb (C), gait angle (degrees; D), and total mobility (horizontal + vertical) in an open field on P31 (E). (TIF) [file pone.0047499.s003.tif]

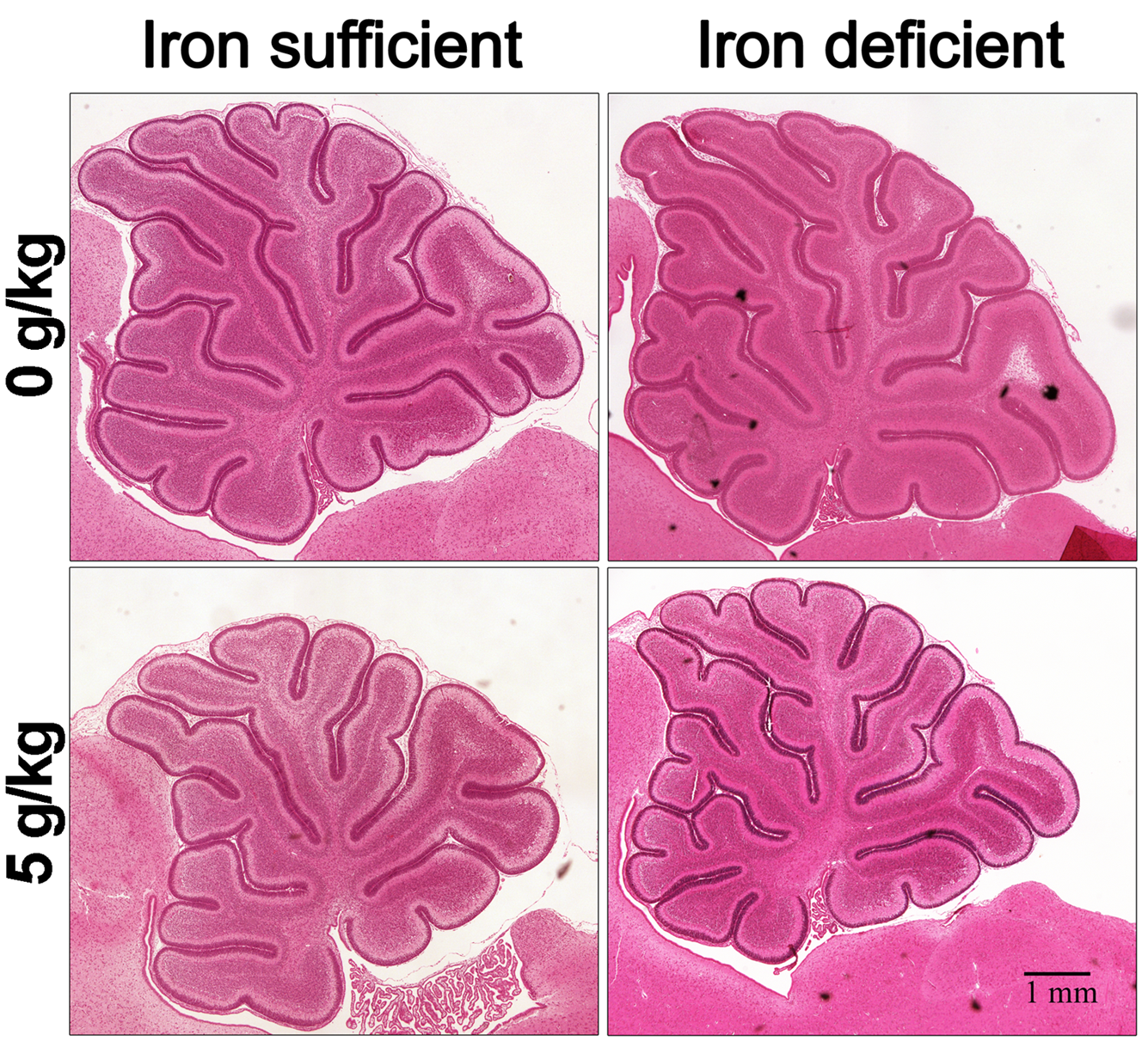

Supplement: Figure S4 — Cerebellar morphology. Although alcohol-exposed pups (5 g/kg) had smaller cerebella, overt cerebellar morphology was normal in alcohol-treated offspring of IS and ID dams. (TIF) [file pone.0047499.s004.tif]
