# Supplementary material for: Adequacy of Maternal Iron Status Protects against Behavioral, Neuroanatomical, and Growth Deficits in Fetal Alcohol Spectrum Disorders
Source: PLoS One. 2012 Oct 19;7(10):e47499. doi: 10.1371/journal.pone.0047499 (PMC3477151; doi:10.1371/journal.pone.0047499)
Supplement: Table S1 — Dam Iron Status. Blood and liver values at P5 and P22 for IS and ID dams used in this study. (DOC) [file pone.0047499.s005.doc]

|  | **Table S1. Dam Iron Status** | | | | |
| --- | --- | --- | --- | --- | --- |
|  | Reference | Postnatal Day 5 | | Postnatal Day 22 | |
|  | Range^†^ | Iron sufficient | Iron deficient | Iron sufficient | Iron deficient |
| Red blood cell number, x 10^6^/μL | 6.5–9.2 | 7.5±0.1(6) | **7.1±0.1(7)*** | 8.1±0.1(6) | 7.7±0.4 (7) |
| Hemoglobin, g/dL | 14-17 | 14.8±0.1(6) | **13.1±0.1(6)*** | 16.2±0.3(6) | **14.8±0.5(7)*** |
| Hematocrit, % | 36-52 | 41±1(7) | **37±1(8)*** | 45±1(7) | 43±1(7) |
| Mean corpuscular volume, fL | 50-60 | 56±1(7) | **53±1(8)*** | 57±1(6) | **54±1(7)*** |
| Mean corpuscular hemoglobin, pg | 16-20 | 20.0±0.2(7) | **19.0±0.2(7)*** | 19.9±0.2(6) | **18.9±0.4(7)*** |
| Red cell distribution width, % | 10.6-14.6 | 13.2±0.2(6) | **15.8±0.3(6)*** | 13.2±0.1(7) | **15.7±0.4(7)*** |
| Serum Fe, μg/dL | 137-380 | ND | ND | 438±25(8) | **328±35(6)*** |
| Total iron binding capacity, μg/dL | na | ND | ND | 557±28(9) | 560±17(7) |
| Transferrin Saturation, % | na | ND | ND | 84±5(9) | **57±6(7)*** |
| Liver Fe, ppm | na | ND | ND | 165±35(10) | **65±11(7)*** |

**†** Mean control range of non-pregnant, adult female CD or Crl:WI(Han) rats^1-5^. *****, significantly different, *P* < 0.05, from iron sufficient dams at the same time point using mixed model analysis. Mean ± SEM is shown. Numbers in parens indicate the number of animals examined for each measure. na, not available; ND, not determined.

[1] Giknis ML, Clifford CB. (2008) Clinical laboratory parameters for crl:WI(han)*. Charles River Laboratories*. [2] LaBorde JB*, et al.* (1999) Haematology and serum chemistry parameters of the pregnant rat*. Lab Anim* 33:275-287. [3] Levine DS (1995) *CRC Handbook of Toxicology,* eds Derelanko MJ, Hollinger MA. CRC Press:Boca Raton. pp 517-537. [4] Cerven D, DeGeorge G, Bethell D. (2008) 28-day repeated dose oral toxicity of recombinant human holo-lactoferrin in rats*. Regul Toxicol Pharmacol* 52:174-179. [5] Car BD, Eng VM, Everds NE, Bounous DI. (2006) *The Laboratory Rat,* eds Suckow MA, Weisbroth SH, Franklin CL. Academic Press:Boston, p 132.
