# Supplementary material for: Adequacy of Maternal Iron Status Protects against Behavioral, Neuroanatomical, and Growth Deficits in Fetal Alcohol Spectrum Disorders
Source: PLoS One. 2012 Oct 19;7(10):e47499. doi: 10.1371/journal.pone.0047499 (PMC3477151; doi:10.1371/journal.pone.0047499)
Supplement: Table S2 — Pup Iron Status. Blood and liver values for IS and ID pups at P10 and P35 that were gavaged with 0, 3.5, or 5.0 g/kg alcohol daily from P4 to P9. (DOC) [file pone.0047499.s006.doc]

|  | | **Table S2. Pup Iron Status** | | | | | |
| --- | --- | --- | --- | --- | --- | --- | --- |
|  | | **Iron sufficient** | | | **Iron deficient** | | |
| **Alcohol dose, g/kg/day** | | **0** | **3.5** | **5** | **0** | **3.5** | **5** |
| Red blood cell  number, x 10^6^/μL | P10 | 3.2±0.1(7) | 3.1±0.1(7) | 3.0±0.1(5) | 2.9±0.2(10) | 2.9±0.1(6) | 3.0±0.1(6) |
|  | P35*^†^ | 5.8±0.1(5) | 5.9±0.1(5) | 5.6±0.1(7) | 5.9±0.1(6) | 6.2±0.2(5)^§^ | 5.8±0.2(6) |
| Hemoglobin, g/dL | P10* | 9.5±0.5(7) | 9.0±0.2(7) | 9.3±0.2(5) | 7.2±0.4(9)^§^ | 7.8±0.5(6)^§^ | 8.2±0.2(6)^§^ |
|  | P35 | 12.6±0.1(5) | 12.4±0.1(5) | 12.2±0.2(7) | 12.1±0.2(6) | 12.2±0.4(5) | 12.1±0.2(6) |
| Hematocrit, % | P10* | 29±1(7) | 29±1(7) | 28±1(5) | 23±1(10)^§^ | 25±2(6)^§^ | 26±1(6) |
|  | P35 | 39±1(5) | 37±1(6) | 38±1(7) | 39±0.4(6) | 38±1(5) | 38±1(6) |
| Mean corpuscular  volume, fL | P10* | 92±2(8) | 94±2(7) | 93±1(5) | 78±4(10)^§^ | 84±5(6)^§^ | 86±3(6)^§^ |
|  | P35*^†^ | 68±1(5) | 65±1(6) | 68±1(7) | 66±1(6) | 61±1(5)^‡^ | 66±1(6) |
| Mean corpuscular hemoglobin, pg | P10*^†^ | 28.2±0.3 (8) | 28.6±0.7(7) | 31.2±1.4(5)^‡^ | 24.3±1.1(10)^§^ | 26.3±1.3(6) | 27.1±0.9(6)^§^ |
|  | P35* | 21.8±0.3(5) | 21.0±0.4(6) | 21.7±0.3(7) | 20.5±0.3(6)^§^ | 19.5±0.2(5)^§^ | 21.1±0.3(6) |
| Red cell distribution width, % | P10* | 23.1±1.8(8) | 21.9±0.7(7) | 21.2±1.2(5) | 24.7±1.1(10) | 25.1±2.1(6) | 22.5±0.8(6) |
|  | P35* | 17.1±0.7(5) | 20.1±1.2(6) | 16.5±0.5(7) | 24.8±0.7(6)^§^ | 22.9±1.7(5) | 22.5±0.7(6)^‡§^ |
| Serum Fe, μg/dL | P10* | 188±16(8) | 162±13(7) | 198±16(6) | 95±17(12)^§^ | 136±25(12) | 126±20(6)^§^ |
|  | P35 | 262±29(6) | ND | 237±24(9) | 266±28(8) | ND | 233±23(7) |
| Total iron binding  capacity, μg/dL | P10 | 466±26(8) | 428±37(7) | 501±15(6) | 519±21(12) | 506±21(12) | 515±15(5) |
|  | P35 | 431±16(6) | ND | 437±13(9) | 454±23(8) | ND | 410±9(7) |
| Transferrin Saturation, % | P10* | 42±5(8) | 40±5(7) | 40±4(6) | 19±4(12)^§^ | 28±6(12) | 21±2(6) |
|  | P35 | 62±7(6) | ND | 54±5(9) | 59±6(8) | ND | 57±5(7) |
| Liver Fe, ppm | P10*^†¶^ | 42±4(11) | 52±7(7) | 93±22(9)^‡^ | 19±2(10)^§^ | 16±1(10)^§^ | 20±4(8)^§^ |
|  | P35* | 135±11(11) | 152±17(5) | 165±16(8) | 176±14(13)^§^ | 180±20(6) | 191±9(12) |

**Supplemental Table 2. Pup iron status.** Parens indicates the number of animals examined for each measure. ND, not determined. Symbols indicate main effect of diet (*) and alcohol (†) at that time point. ‡, significantly differs from 0 g/kg within that Diet group. §, significantly differs from iron-sufficient at the same alcohol dose. ¶, significant Diet x Alcohol interaction at this age.
